# An Innovative Strategy for Achieving Interface Gradient Material Using Co-Deposition Technology

**DOI:** 10.3390/nano15100718

**Published:** 2025-05-09

**Authors:** Yanxin Zhang, Liyan Lai, Yan Luo, Zhuoqing Yang, Guifu Ding

**Affiliations:** 1 State Key Laboratory of Micro-Nano Engineering Science, Shanghai Jiao Tong University, 800 Dongchuan Road, Shanghai 200240, China; zhangyx2019@sjtu.edu.cn (Y.Z.); yzhuoqing@sjtu.edu.cn (Z.Y.); 2 School of Science, Shanghai Institute of Technology, Shanghai 201418, China; 3 Shanghai Aerospace Electronic and Communication Equipment Research Institute, Shanghai 201109, China; luoyan_tg@163.com

**Keywords:** gradient structure, interface, microstructure, SiC whisker, simulation, coefficients of thermal expansion, co-deposition

## Abstract

In this study, high-performance SiC whisker (SiCw)-reinforced Cu matrix functionally graded materials (FGMs) were achieved through the synergy of numerical simulation and co-electrodeposition and then successfully applied as the interface of Cu and Si. A comprehensive numerical simulation framework was developed to investigate the influence of gradient transition modes and the maximum volume fraction of SiCw on the thermal–mechanical properties in the different gradient structures. The optimized FGMs via numerical simulation were fabricated using a co-electrodeposition technique, producing a 100 μm thick coating with a SiCw volume fraction gradient ranging from 0% to 40%. The interface of Cu and SiC was void free in the FGMs and the SiCw was coated by nano-scale Cu grains during the electroplating process. The coefficient of thermal expansion in FGMs was higher than 9.78 × 10^−6^ K^−1^, which was the coefficient of thermal expansion in the Cu-40% vol. SiCw, and lower than 16.4 × 10^−6^ K^−1^, which was the coefficient of thermal expansion in pure Cu. Notably, the bonding interface area between the Cu/Si joint with the gradient-structured FGMs was more than twice that of non-graded materials. The enhanced thermal–mechanical performance was attributed to the synergistic effects of the nano-scale grain-reinforced SiCw-Cu interface and an optimized stress distribution achieved by the gradient structure.

## 1. Introduction

With the continuous development of 5G communication technology and high-performance supercomputers, the size of chips has decreased, and the density of integrated circuit has increased accordingly. Recently, research has shown that the power density of micro-electronics has reached 1000 W/cm^2^ [1]. Due to the high-power density and heterogenous distribution of the heat source for the chips, heat tended to accumulate at the local area and led to the formation of hotspots [2]. The existence of the hotspots during the working process was deleterious to the stability of the chips [3]. Therefore, rapidly decreasing the temperature of the hotspots was critical to ensuring the reliable performance of the device service.

During the heat dissipation process, extreme heat accumulation and residual thermal stress easily occurred at the interface between the semiconductor chip and the metal heat sink because of the significant difference in their coefficients of thermal expansion (CTEs) and mechanisms of heat transfer. Owing to the high conductivity and excellent machinability, copper (Cu) was an acceptable material to act as the interface material between the chip and the heat sink [4,5]. However, the applications of pure Cu were limited by its disadvantages of low tensile strength and high corrosion rate [6]. The traditional strategy to improve the properties of Cu was to introduce the reinforcements, including the SiC, SiO_2_, CNT, and Al_2_O_3_ particles. Among these reinforcements, SiC stood out as an exceptionally promising material with the properties of wide band gap (2.39–3.33 eV), high modulus (Young’s modulus reached 424 GPa), low thermal expansion, high thermal conductivity (single crystals reach 490 W/m·K), and excellent chemical stability [7,8,9,10]. The SiC-reinforced Cu matrix composites were the optional selections to solve the problem of residual stress [11], while the major drawback of composite materials was that their properties remain unchangeable due to the homogeneous composition [12,13].

Functionally graded materials (FGMs) can resolve these problems by reconstruing and fabricating the composition [14]. Due to the variation in structure or composition over the volume gradually, the properties of FGMs were distinctive from individual materials. For the FGMs, the hybrid interfaces among the composites and the base materials were exchanged into smooth transition with gradually changing interface, and the FGMs had resistance to failure at the sharp hybrid interface [15]. Functionally graded copper matrix composites reinforced by SiC are attractive materials for a broad range of engineering applications with superior thermal–mechanical properties [14].

There were many manufacturing technologies to fabricate FGMs, consisting of centrifugal casting, sintering, powder metallurgy, electrodepositing, etc. [14,15,16]. The conventional process, involving high-temperature and high-pressure treatments, often leads to the failure of the mechanical structure and the degradation of the SiC [14]. With these conditions, Cu and SiC reacted easily, forming excessive carbide with high thermal resistance at the interface. In addition, substantial residual stress was generated at the interface, potentially leading to the formation of microcracks, owing to the high-temperature processing and quite different CTE between the metal and SiC [15]. These problems could be mitigated by low-temperature manufacturing techniques such as electroplating, cold spray, and vapor deposition [16]. Among these techniques, electroplating technology could suppress the agglomeration of SiC and fabricate micro- to millimeter-scale material with the advantages of low coat and the precise control of the layer thickness.

It was reported that SiC-reinforced Cu matrix FGMs with five layers were fabricated by changing the electrodeposition parameters, such as the current density and stirring speed [17]. Compared with pure copper and single-layer Cu-SiC MMCs with the same thickness, the corrosion and wear efficiency of FGMs was significantly reduced [18,19]. However, research on the SiC-reinforced Cu matrix FGMs mainly focuses on SiC particles. Research on whisker (SiCw)-reinforced Cu matrix FGMs is poor. SiCw was also an acceptable strengthening phase material for Cu matrix composites [20]. Li et al. reported that SiCw could improve the tensile strength of the Cu matrix, and the SiCw-reinforced Cu matrix composites also had a thermal conductivity of 312 W/m·K [21]. During the tensile test, the whisker in the composites could suppress crack propagation and bear part of the load which was applied at the matrix [22]. The SiCw has the potential to be applied to Cu matrix FGMs.

In this study, high-performance SiCw-reinforced FGMs were manufactured using electroplating technology. First, the finite element method was employed to systematically study the effects of gradient-transition modes and the contents of whiskers on mechanical properties. Subsequently, the designed structure SiCw/Cu FGMs were prepared and studied through microstructure analysis. Finally, the FGMs were used as an interface layer to bond Si and Cu.

## 2. Experiments and Simulation

### 2.1. Experiments and Characterization

**Figure 1:**
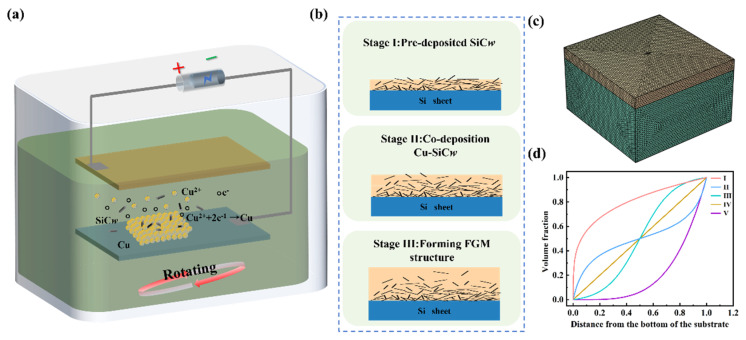
The schematic diagram of the (**a**) co−electroplating equipment and the (**b**) manufacturing process of FGMs. (**c**) The simulation models of and (**d**) the transition mechanism of the FGMs.

The SiCw-reinforced Cu matrix FGMs were manufactured by co-electrodeposition and the schematic diagram is shown in Figure 1a. A copper (Cu) plate (99.9% purity) with the size of 100 mm × 50 mm × 2 mm was set as the anode and the cathode was a 20 mm × 20 mm × 1 mm titanate (Ti) sheet. The distance between cathodes and anodes was 20 mm. Before the co-electrodepositing process, the Cu plate and Ti sheet were polished by 2000 # sand paper to remove the oxide film at the surface and then cleaned by the deionized water. The electrodes were in parallel to each other and perpendicular to gravity direction in order to eliminate the influence of gravity on the quality of the FGMs. The β-SiCw (XFNANO Material Co., Ltd., Nanjing, China), with a length of 10~50 μm and diameter of 3~10 μm, was cleaned by the 3 vol.% hydrofluoric acid and then ultrasonically washed in distilled water until the pH become neutral. The cleaned SiCw was added into the electroplating bath and stirred by a continuous magnetic rotator for 8 h. The performance characterization of SiCw has been shown in our previous work [23] and in Figure S1. The detailed components of the bath are shown in Table S1. The content of SiCw in the bath was 1 g/L, the current density was 10 mA/cm^2^, and the temperature of the bath was 25 °C.

To achieve different components of SiCw reinforcement Cu matrix composites, the magnetic stirring equipment was employed and the rotating speeds of 0 rpm, 100 rpm, 200 rpm, 300 rpm, and 400 rpm were applied for 6 h. Based the results of the effects of rotating speeds on the coating microstructure, FGMs were fabricated in three stages as shown in Figure 1b. At the pre-deposited stage, the Si sheet sputtered by Cr/Cu was used as the cathode and put into the electrolyte. The rotating speed was set at 0 rpm, and the deposition time was about 20 min. During the co-depositing process, the rotating speed of 100 rpm was applied for 1 h. Then, the rotating speed was increased to 400 rpm at a rate of 50 rpm per hour. After this process, co-deposition was carried out at a rotation speed of 400 rpm for 3 h. Finally, the coating was put into a pure Cu bath and deposited for more than 3 h to form a pure Cu surface.

The morphology of the samples was observed on a Rise–Magna field-emission scanning electron microscope (SEM, Brno, Crech) with 15 kV working voltage. In addition, the elemental mapping was obtained by the associated energy-dispersive spectroscopy (EDS, Ulm, Germany). The thermal deformation was carried by a high-temperature annealing furnace (Shengli Instruments, Shanghai, China) and then the variation in the surface morphology was measured by a VHX-7000 3D optical microscopy (OM, Osaka, Japan). The CTE of the samples was measured by a thermal dilatometer (DIL 402 Expedis, Selb, Germany). The sample for the CTE test was in the dimension of 32 mm × 2 mm × 0.1 mm. The hardness and the elastic modulus of the samples were calculated from the keysight G200 nano-indentation test. The bonding process was carried by a muffle furnace with N_2_ as protective gas. The shear test of joints was carried by a bonding strength tester (PTR-1101, Osaka, Japan) for 3 samples at each condition.

### 2.2. Numerical Methodology

To optimize the structure of FGMs, numerical simulation models were established using COMSOL Multiphysics 5.4 finite element simulation software. The simulation model consisted of a FGMs (1000 μm × 1000 μm × 100 μm) and Si sheet (1000 μm × 1000 μm × 500 μm) as shown in Figure 1c. The model was heat from 273 K to 373 K to study the stress distribution under different gradient-transition modes, which is shown in Figure 1d. The continuous transition material was divided into different layers, whose mechanical properties were obtained by the simple mixing rule. By altering the transition mechanism of component variation in SiCw, the mechanical properties of the FGMs were obtained. Based on these results, FGMs with varying maximum volume fractions of SiCw were also studied.

## 3. Results and Discussion

### 3.1. Deformation of the FGMs by Numeric Simulation

**Figure 2:**
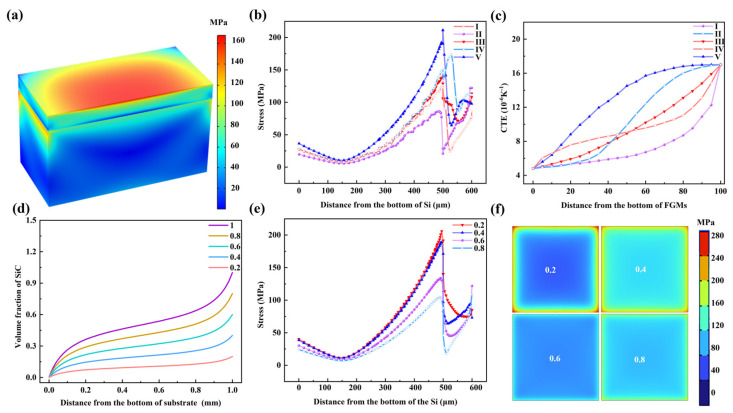
(**a**) Stress distribution of the Si sheet coated FGMs; (**b**) stress along the thickness direction and (**c**) CTE along the thickness; (**d**) the gradient mode with different SiCw content; (**e**) stress along the thickness direction; (**f**) stress distribution of the FGMs/Si interface.

To facilitate the observation of the deformation behavior of FGM-coated Si, the deformed model was cut along the symmetry plane, and the stress distribution is shown in Figure 2a. The stress was mainly concentrated in the FGMs and the region near the Si/FGMs interface. The interface strength between Si and FGMs was critical to the structural performance. Therefore, typical regions, such as curves I, were selected for further analysis of the stress behavior, as shown in Figure 2b. The stress along curve I, starting from the bottom of Si, initially increased, then decreased, and finally increased again. The first stress peak appeared at the Si/FGMs interface, while another stress peak was observed on the surface of the FGMs. Additionally, the stress at the Si/FGM interface was higher than that on the FGM surface. The Si/FGM interface serves as a transition zone between two materials, where the bonding strength is influenced by the differences in physical properties between metals and ceramics. Therefore, reducing stress is crucial to ensuring interface stability.

For different gradient transition modes, the stress at the interface was lower under the second gradient than with others. To further understand the stress reduction, the variation in the CTE for different gradient modes was analyzed, as shown in Figure 1c. Using Gradient II resulted in a thicker low-CTE region within the FGMs, which alleviated the thermal mismatch between Si and the FGMs near the interface, thereby reducing stress. The second gradient in FGMs effectively optimized the transition behavior and improved the residual stress distribution.

In addition to the gradient type, the volume fraction of SiCw in the FGMs also played a critical role on the stress distribution. To investigate this issue, a Si/FGM model with varying SiCw volume fractions (Figure 2d) was studied. With the volume fraction of SiCw ranging from 100% to 20%, the stress along curves I increased, as shown in Figure 2e. When the SiCw volume fraction was 80%, the peak stress was located on the FGMs surface but as it decreased to 60%, the peak stress shifted to the Si/FGM interface and remained there as the volume fraction further reduced. This result indicated that the SiCw content not only altered the stress magnitude but also altered its distribution. Given the significant change in material properties at the Si/FGMs interface, particular attention is needed for stress distribution there and the stress distribution at the Si/FGM interface is investigated in Figure 2f. With the volume fraction decreased from 80% to 20%, the maximum stresses at the interface were 124 MPa, 166 MPa, 217 MPa, and 283 MPa, respectively.

The contents of SiCw strongly dominate the stress and the FGMs with high contents of SiCw was helpful to reduce the thermal stress at the Si/FGM interface. However, it was deleterious to the properties of the composites with a high content of reinforcement owing to the hugely different properties between the matrix and reinforcements [24]. In addition, there were few reports for the Cu/SiCw composites with the SiCw volume fraction more than 60%. Generally speaking, the FGMs with the second gradient and the maximum volume fraction of SiCw in 40% were the ideal and machinable structure material to ameliorate the stress distribution.

### 3.2. Microstructure and Morphology of the FGMs

#### 3.2.1. Homogeneous Cu/SiCw Coating

**Figure 3:**
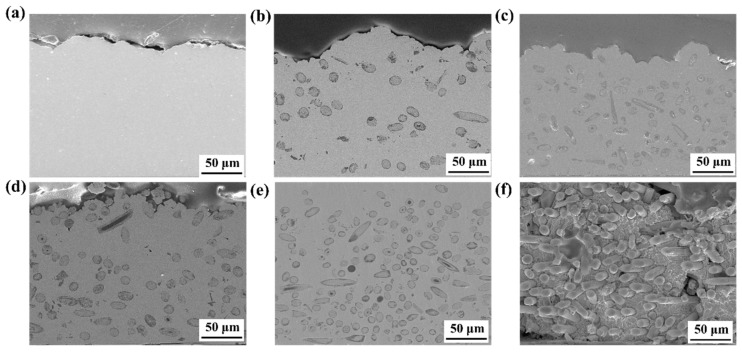
The Cu/SiCw composites with SiCw volume fraction of (**a**) 0, (**b**) 10%, (**c**) 20%, (**d**) 30%, (**e**) 40%, and (**f**) 60%.

The Cu/SiCw composites with different SiCw volume fractions were fabricated using electroplating technology shown in Figure 3. The SiCw volume fraction varied from 0% to 40%. The pure Cu was uniform and dense, as shown in Figure 3a. With the low content of SiCw, the bonding of SiCw and the matrix was compact and the distribution of the SiC was irregular for volume fractions not exceeding 38%. When the volume fraction of SiCw reached 60%, the void defects appeared at the surroundings of the whisker and the matrix became coarse and loose, which was detrimental to the properties of the composites.

During the electroplating process, the coating material was influenced by the electroplating parameters [25], the contents of SiCw [26], and the external applied energy field [27]. In this study, the primary variable was the rotation rate and SiCw volume fraction. With the high rotating rate of 600 rpm in SiCw free bath, pure Cu without defect was manufactured, indicating that the electroplating bath was stable and the electroplating parameters were optimal. The results shown in Figure 3b–f were obtained with the same content SiCw of 1 g/L. With the rotating rate of 600 rpm, 400 rpm, 300 rpm, 200 rpm, and 0 rpm, the Cu/SiCw composites with the SiCw volume fraction of 10%, 19%, 28%, 38%, and 60% were fabricated. With the decreasing of the rotating rate, the motion velocity of the SiCw slowed down and the viscous force of electroplating solution acting on the whiskers reduced. Owing to the existence of gravity, the deposition rate of SiCw increased with the decreasing of rotating rate. Additionally, the ion exchange behavior became weak at the lower rotation rate. At high rotating rates of 400 rpm and 600 rpm, the depositing rate of SiCw was low and the sufficient ion exchange was performed. When the rotating rate reduced to 300 rpm, micro voids began to appear at the surrounding of the SiCw owing to the insufficient ion exchange behavior. With the rotating rate of 0 rpm, the composite with SiCw volume fraction of 60% was fabricated. There were many voids and inhomogeneous matrix microstructures in the composites. Therefore, the SiCw volume fraction of the composites should not exceed 40%.

#### 3.2.2. Microstructure and Crystallographic Characteristics of FGMs

**Figure 4:**
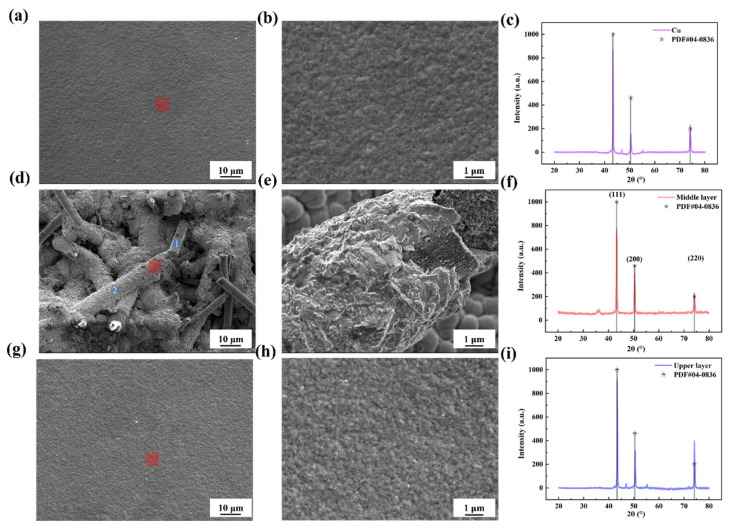
The surface of the FGMs and pure Cu. (**a**) The SEM image of pure Cu and the enlarged area of red squareness (**b**). (**c**) The XRD results of pure Cu. (**d**) The SEM image of pre-deposited coating and the enlarged area of red squareness (**e**). (**f**) The XRD results of pure Cu. (**g**) Surface morphology of FGMs and the enlarged area of red squareness (**h**). (**i**) The XRD results of FGMs.

Based on experiment results and the optimized numerical model, the Cu/SiCw FGMs were manufactured with SiCw volume fractions varying from 0% to 40%. Figure 4 dictated the surface morphology of the FGMs and pure Cu. The surface of the pure Cu was dense (Figure 4a) with grain size lower than 1 μm (Figure 4b). For the pre-deposited coating shown in Figure 4d,e, the whiskers were coated with electroplated Cu, indicating that the Cu coating closely adhered to the whisker. After the co-depositing process, the surface was flat with no void occurring at the coating (Figure 4g,h). To further study the grain behavior of the FGMs, XRD, and EDS tests were conducted. The XRD results in Figure 4c showed three distinctive characteristic peaks occurred at 43.3°, 50.4°, and 74.1°, corresponding to (111), (200), and (220) crystal planes of metallic Cu in PDF # 04-0836. The characteristic peaks in Figure 4f and Figure 4i were consistent with those in Figure 4c. From the XPS results in Figure S2, there were O-based bonds (O 1s peak), C-based bonds (C 1s peak), Si-based bonds (Si 2p peak), and Cu-based bonds (Cu 2p3) in the coating. Therefore, the coating consisted of Cu and SiC. From the EDS testing in Table 1, the rod-like structure was SiCw and the coating mainly consisted of Cu, shown in Figure 4d. To study the interface of Cu/SiCw further, the TEM image is shown in Figure S3. The interface between the Cu/SiCw interface was void-free. The element analysis showed that there was no element diffusion behavior at the interface. The fast Fourier transform pattern in Figure S4 shows that the interface was incoherent. These results indicated that SiCw had a good interface with Cu coating.

**Figure 5:**
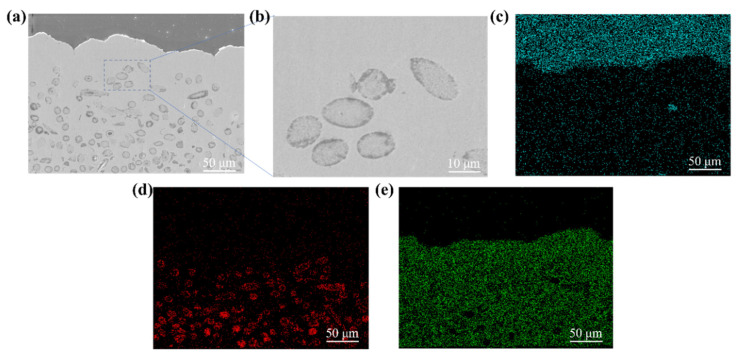
(**a**) The SEM image of the FGM and the enlarged area of the rectangle (**b**). The elements distribution of (**c**) C, (**d**) Si, and (**e**) Cu of the FGM.

To further clarify the microstructure of FGMs, the cross-section of the FGMs is shown in Figure 5. SiCw was densely distributed at the bottom of the material, with its density decreased along the thickness direction. From the magnified image with the square frame in Figure 5a, the interface between Cu and SiCw was intact and void-free (Figure 5b). To investigate the distribution behavior of SiCw in the FGMs, the element distribution of C, Si, and Cu are displayed in Figure 5c–e. Due to the existence of C element in the test environment, the distribution of C in the FGMs was irregular and the relationship between SiCw and the C element was independent. The distribution of Si in Figure 5d is in accordance with that of SiCw in Figure 5a.

To facilitate the comparison between the experimental result and the simulation results, the FGMs were divided into five layers, denoted as 0.2, 0.4, 0.6, 0.8, and 1.0, to represent the transition from the pure Cu region to the region with the highest volume fraction.

**Figure 6:**
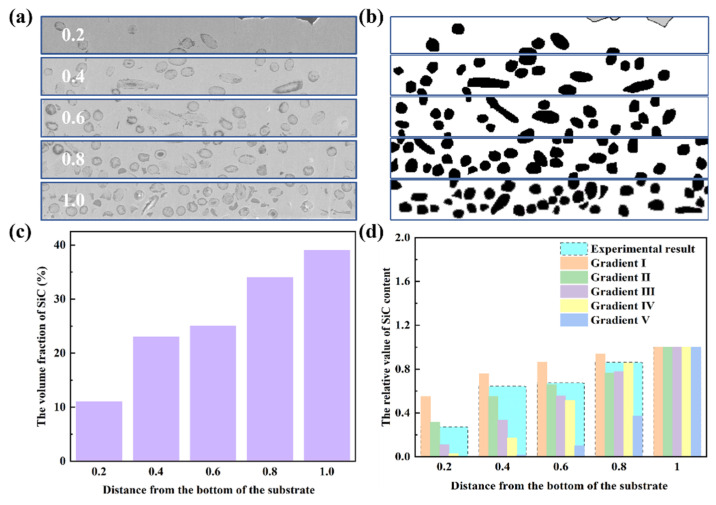
(**a**) The five divided SEM images of the FGMs and the (**b**) gray processed image. (**c**) The volume fraction of SiCw in different layers. (**d**) The component comparison of experiment and simulation results.

The processed pictures are shown in Figure 6a. In this study, the volume fraction of SiCw was calculated by counting the pixels of SiCw and the results are displayed in Figure 6b. The volume fraction of SiCw in the different layers was 11%, 23%, 25%, 34%, and 39% with the contents of SiCw increasing shown in Figure 6c. After the normalization treatment, the relative volume fraction of low content SiCw to the highest contents SiCw was 27%, 60%, 64%, 84%, and 100%. Compared to the result with the five kinds of FGMs in Figure 6d, the experimental result approached the FGMs with the second gradient. The experimental result was in accordance with the optimized model.

### 3.3. The Thermal and Mechanical Properties of the FGMs

**Figure 7:**
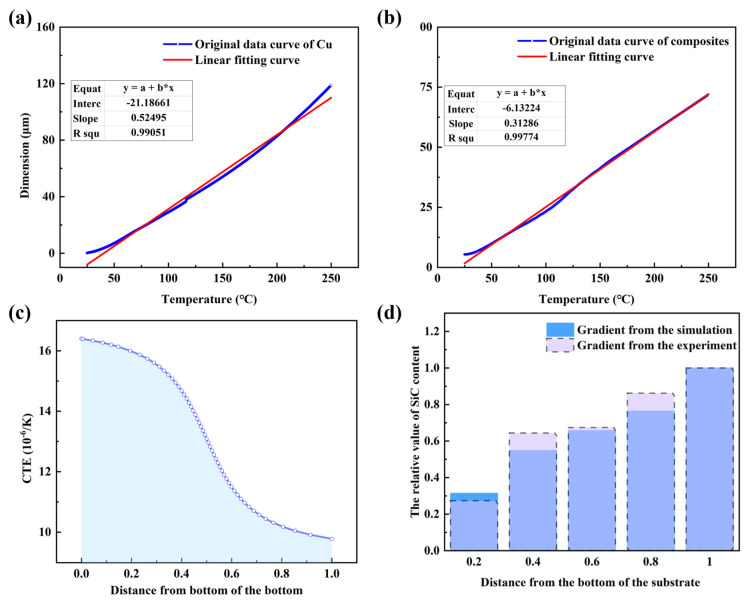
The thermal results of (**a**) the composites and (**b**) pure Cu. (**c**) The variation in the CTE using the simulation model. (**d**) The relative value of SiCw content in the experiment and the simulation.

For the Cu/SiCw FGMs, the CTE was hard to measure owing to the fact that the deformation along the gradient direction was heterogeneous during the heating process. Therefore, the CTE of FGMs was studied by combining the thermal dilatometer testing and the simulation model in this study. The CTE of composites with the volume fraction of SiCw in 40% and pure Cu is shown in Figure 7a,b. After the linear fitting, the CTE of composites and Cu was 16.4 × 10^−6^ K^−1^ and 9.78 × 10^−6^ K^−1^. Considering that the gradient structure in this study was similar to the second transitional model, the he CTE distribution along the gradient direction could be exposed in Figure 7c. For the FGMs, the CTE along the gradient direction decreased quickly firstly, and then decreased slowly. By integrating shadow part, the mean CTE of the FGMs was 12.85 × 10^−6^ K^−1^. Considering the difference in the simulation model and the experiment result, the mean CTE of the FGMs was 12.12 × 10^−6^ K^−1^. Therefore, the FGMs realized the transition of CTE from 16.4 × 10^−6^ K^−1^ to 9.78 × 10^−6^ K^−1^ with the optimized structure in micro-scale.

**Figure 8:**
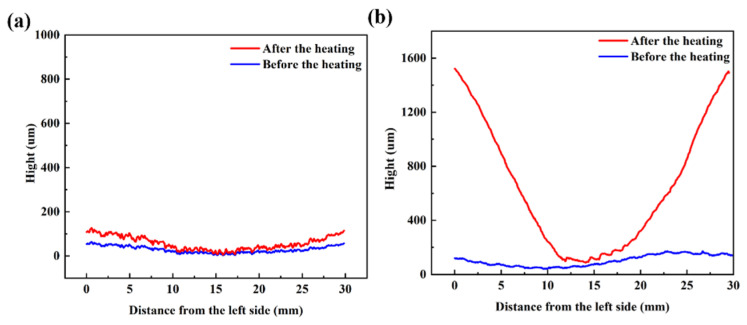
The deformation behavior of (**a**) pure Cu and (**b**) FGMs before and after heating process.

To further study the deformation behavior of the FGMs with the thermal load, the FGMs and pure Cu samples, with the size of 32 mm × 2 mm × 0.1 mm, were heated to 260 °C, and then cooled down to room temperature. The deformation behaviors of pure Cu and FGMs samples are shown in Figure 8a,b. There was almost no bending for the pure Cu after heating while the FGMs bent up along the gradient direction. The height variation in Cu was lower than 200 μm while that of FGMs reached 1460 μm. The bending of coating may be related to the residual stress owing to the unbalanced crystallization during the electroplating process [28]. During the heating process, the residual stress was released, and the coating could deform with the redistributed stress. However, the deformation of Cu was much lower than that of FGMs. Therefore, the bending behavior of the FGMs was dominated by the gradient structure. Plastic deformation occurred during the heating process and bending behavior continued after heating.

**Figure 9:**
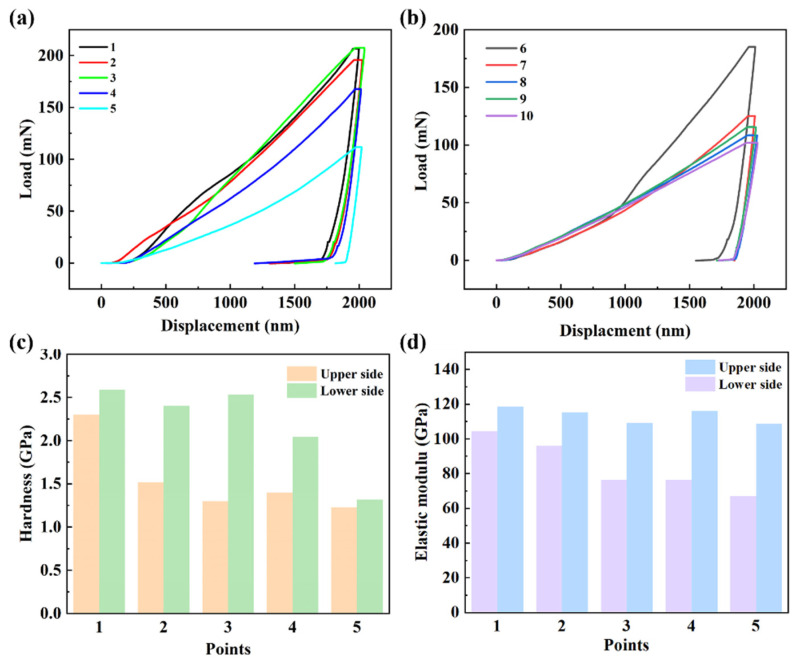
The load–displacement at the (**a**) upper layer and the (**b**) lower layer. (**c**) Hardness and (**d**) Elastic modulus for the FGMs. (This picture has been corrected).

**Figure 10:**
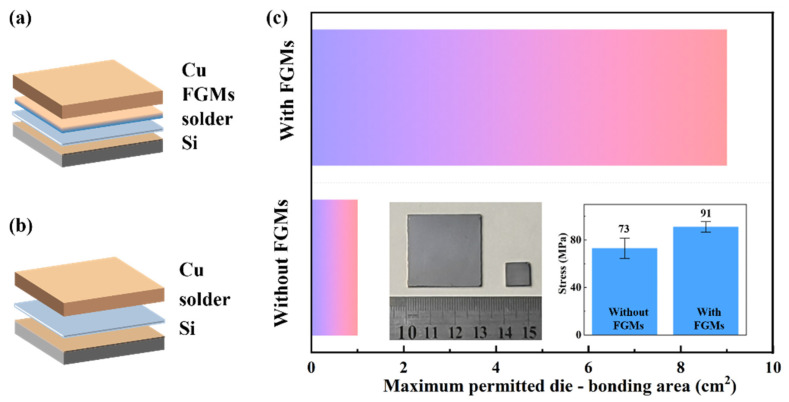
The bonding structure of Cu and Si (**a**) without and (**b**) with FGMs as interface. (**c**) Maximum permitted die-bonding area. This picture has been corrected.

The nanoindentation test was applied to study the properties of samples including the hardness and elastic modulus (Figure 9). The load–displacement curve at the upper layer with low content SiCw and the lower layer with the high content SiCw is shown in Figure 9a,b. The displacement under holding stages was related to the creep behavior [29]. The mean hardness at the lower layer of 2.18 GPa was much higher than that at the upper layer shown in Figure 10c. In addition to the hardness, the mean elastic modulus at the lower layer of 113.56 GPa was also higher than that at the upper layer of 84.06 GPa. This phenomenon was related to the reinforcement effect of SiCw on the Cu matrix, including the fine grain strengthening and second phase strengthening [30]. Compared with the pure Cu, the FGMs improve the mechanical properties, including the elastic modulus and hardness at the same time.

To qualitatively describe the improvement effect of FGMs on the interface bonding, the bonding behavior of Cu and Si with/without FGMs as an interface in different sizes is shown in Figure 10. The structures of the Cu/Si joint and Cu/FGMs/Si are shown in Figure 10a,b. The shear strength of joints with/without FGMs was performed at room temperature. Without the FGMs, the shear strength of the joint, which consisted of Cu, solder, and Si, was 73 MPa. However, the shear strength of the joint with the FGMs was 91 MPa, which was 24.5% higher than that without FGMs. For the joint without the FGMs, the maximum permitted die-bonding area was 1 cm^2^. However, the maximum permitted die-bonding area reached 9 cm^2^ with the FGMs as the interface shown in Figure 10c. The FGMs could realize about eight times bonding zone than that without FGMs, due to the optimized gradient structure. 

## 4. Conclusions

This work presented a novel strategy to obtain interface gradient material through co-electrodepositing technology and simulation. Through the numerical optimization of five transition modes, the second gradient transition modes could decrease the residual stress and ameliorate the distribution of the stress synchronous effectively. Compact and sound SiCw-reinforced Cu matrix FGMs were fabricated with the designed structure, featuring with a SiCw volume fraction ranging from 0% to 40%. Comprehensively considering the experimental and simulation results, the mean CTE of the FGMs was 12.12 × 10^−6^ K^−1^. After the heating process, the FGMs bend up due to the CTE gradient. The mean E at the upper layer of the FGMs was 84.06 GPa and that at the lower layer it was 113.56 GPa. The phenomenon could be related to the grain strengthening and second phase strengthening by the SiCw. The FGMs were successfully applied as the interface material in Cu/Si joints. Owing to their optimized gradient structure, the bonding area of joints incorporating FGMs was more than twice that of joints without FGMs. It has potential for the gradient interlayer to be applied to the high-power device packaging interface, with high-efficiency CTE transition and stress mitigation capabilities.

## Figures and Tables

**Table 1:** The EDS results in different zones.

	Cu	Si	O	C
Point 1	80.7	1.1	4.6	13.6
Point 2	2.8	42.7	11.3	42.2

## Data Availability

Data available on request from the authors.

## Supplementary Materials

The following supporting information can be downloaded at: https://www.mdpi.com/article/10.3390/nano15100718/s1, Figure S1: The performance characterization of SiCw; Figure S2: The TEM images of Cu/SiCw and the EDS mapping in C, Cu and Si; Figure S3: The fast Fourier transform pattern; Figure S4: XPS results of FGMs; Table S1: The detailed components of the bath.
