# Supplementary material for: An Innovative Strategy for Achieving Interface Gradient Material Using Co-Deposition Technology
Source: Nanomaterials (Basel). 2025 May 9;15(10):718. doi: 10.3390/nano15100718 (PMC12114177; doi:10.3390/nano15100718)
Supplement: Supplementary file 1 [file nanomaterials-15-00718-s001.zip › nanomaterials-3553180-supplementary.pdf]

Supplementary

An Innovative Strategy for Achieving Interface Gradient Mate-rial Using Co-Deposition Technology

Yanxin Zhang 1, Liyan Lai 2*, Yan Luo 3, Zhuoqing Yang 1 and Guifu Ding 1*,

1 State Key Laboratory of Micro-Nano Engineering Science, Shanghai Jiao Tong University, 800 Dongchuan Road, Shanghai 200240, China;

2. School of Science, Shanghai Institute of Technology, Shanghai 201418, China;

3. Shanghai Aerospace Electronic and Communication Equipment Research Institute, Shanghai, 201109, China;

* Correspondence: lailiyan312@126.com

* Correspondence: gfding@sjtu.edu.cn

Supporting Notes:

Figure S1: The performance characterization of SiCw

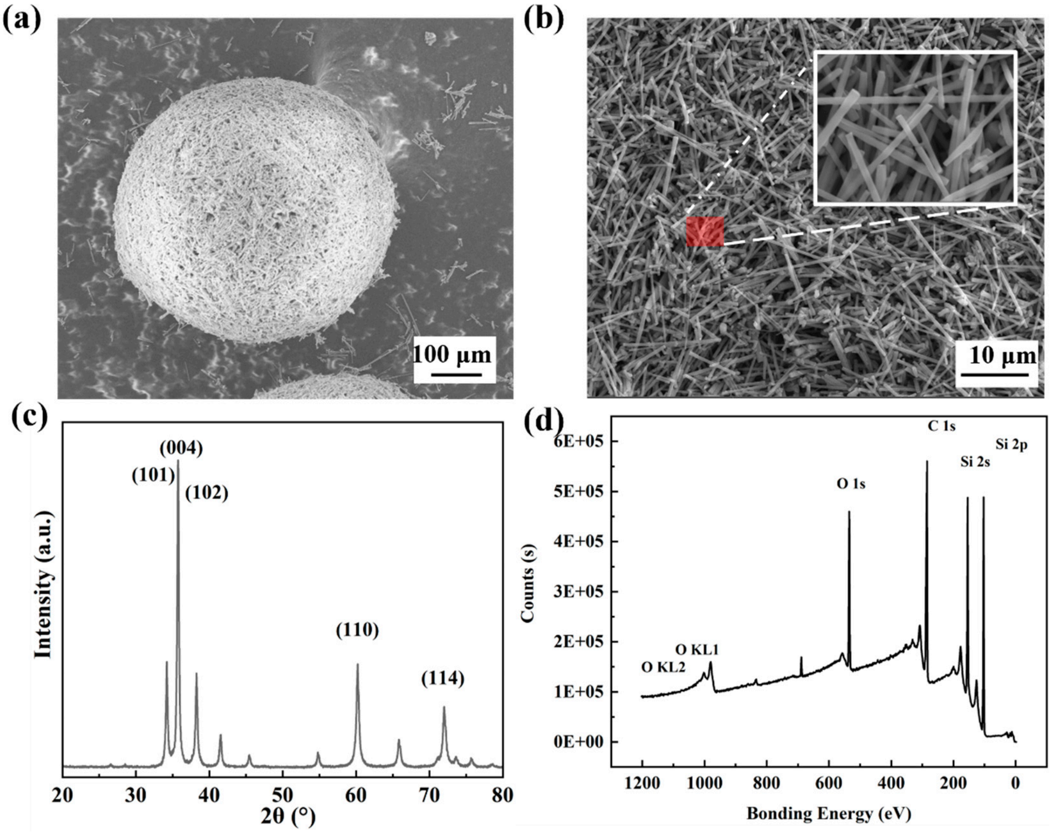

Figure S1. The morphology of SiCw (a) before and (b) after polished. (c) XRD and (d)

It should note that this characterization has been shown in our previous work[1].

Table S1: The detailed components of the bath

Table. S1. Composition and process windows of copper electrolyte.

Composition of bath	Amounts
Cu (CH3SO3)2	80~120 g/L
CH3SO3H	16~24 g/L
Cl-	0.04~0.06 g/L
DVF-B	3~7 g/L
DVF-C	5~10 g/L
DVF-D	4~8 g/L
Applied density	Direct current
Current density	10 mA/cm2
Temperature	25 ℃
SiCw	1g/L

Figure S2: The TEM images of Cu/SiCw and the EDS mapping in C, Cu and Si

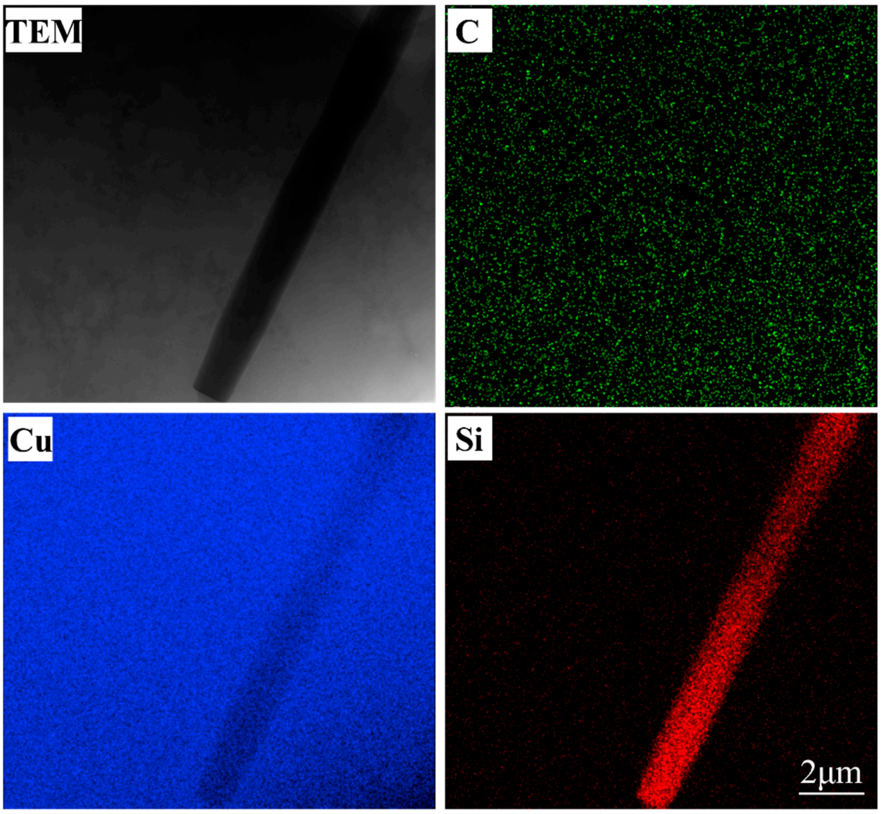

Figure S3: The fast Fourier transform pattern

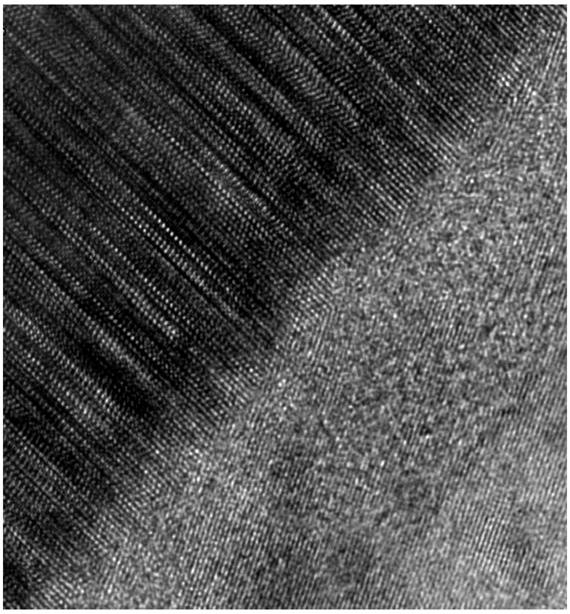

Figure S4: XPS results of FGMs

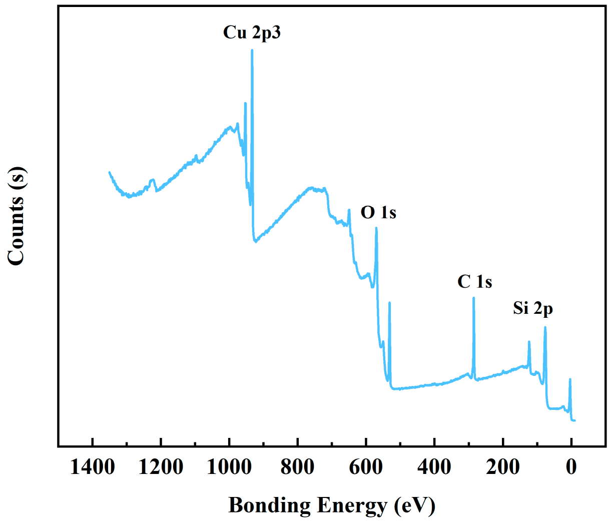

Reference

[1] Y. Zhang, L. Lai, D. Cui, Y. Zhu, H. Cai, B. Yan, Y. Li, Z. Yang, G. Ding, Hybrid effect on mechanical and thermal performance of copper matrix composites reinforced with SiC whiskers, Ceram. Int. 50 (2024) 16553–16563. https://doi.org/10.1016/j.ceramint.2024.02.145.
